# HP1-Mediated Formation of Alternative Lengthening of Telomeres-Associated PML Bodies Requires HIRA but Not ASF1a

**DOI:** 10.1371/journal.pone.0017036

**Published:** 2011-02-15

**Authors:** Wei-Qin Jiang, Akira Nguyen, Ying Cao, Andy C.-M. Chang, Roger R. Reddel

**Affiliations:** 1 Cancer Research Unit, Children's Medical Research Institute, Westmead, New South Wales, Australia; 2 Sydney Medical School, University of Sydney, Sydney, New South Wales, Australia; Texas A&M University, United States of America

## Abstract

Approximately 10% of cancers use recombination-mediated Alternative Lengthening of Telomeres (ALT) instead of telomerase to prevent telomere shortening. A characteristic of cells that utilize ALT is the presence of ALT-associated PML nuclear bodies (APBs) containing (TTAGGG)n DNA, telomere binding proteins, DNA recombination proteins, and heterochromatin protein 1 (HP1). The function of APBs is unknown and it is possible that they are functionally heterogeneous. Most ALT cells lack functional p53, and restoration of the p53/p21 pathway in these cells results in growth arrest/senescence and a substantial increase in the number of large APBs that is dependent on two HP1 isoforms, HP1α and HP1γ. Here we investigated the mechanism of HP1-mediated APB formation, and found that histone chaperones, HIRA and ASF1a, are present in APBs following activation of the p53/p21 pathway in ALT cells. HIRA and ASF1a were also found to colocalize inside PML bodies in normal fibroblasts approaching senescence, providing evidence for the existence of a senescence-associated ASF1a/HIRA complex inside PML bodies, consistent with a role for these proteins in induction of senescence in both normal and ALT cells. Moreover, knockdown of HIRA but not ASF1a significantly reduced p53-mediated induction of large APBs, with a concomitant reduction of large HP1 foci. We conclude that HIRA, in addition to its physical and functional association with ASF1a, plays a unique, ASF1a-independent role, which is required for the localization of HP1 to PML bodies and thus for APB formation.

## Introduction

Alternative Lengthening of Telomeres (ALT) is a telomere length maintenance mechanism that does not involve telomerase [1], [2], and is utilized by many types of tumors including sarcomas and astrocytomas [3]. Although the molecular details of the ALT mechanism in human cells are incompletely understood [4], previous studies have indicated that ALT involves recombination-mediated DNA replication [5], [6]. With a few exceptions [7]–[10], human ALT-positive cells have the hallmarks of (1) a characteristic pattern of telomere length heterogeneity, with telomeres that range from very short to greater than 50 kb long [1], and (2) the presence of ALT-associated promyelocytic (PML) nuclear bodies (APBs) containing (TTAGGG)n DNA and telomere-specific binding proteins [11].

APBs are a subset of PML bodies that are present only in ALT cells, and are not found in mortal cells or telomerase-positive cells [11]. In addition to the constitutive components of PML bodies such as PML and Sp100, telomeric DNA and telomere-associated proteins such as TRF1, TRF2, TIN2 and RAP1 [11]–[13], they also contain other proteins involved in DNA replication, recombination and repair including RAD51, RAD52, and RPA [11], RAD51D [14], BLM [15], [16], WRN [17], BRCA1 [12], MRE11, RAD50, and NBS1 [18], [19], ERCC1 and XPF [20], hRAD1, hRAD9, hRAD17, and hHUS1 [21], FANCD2 [22], Rif1 [23] and hnRNP A2 [24]. Formation of APBs requires NBS1, which recruits MRE11, RAD50 and BRCA1 into these structures [12], [25]. We previously induced APB accumulation with methionine restriction, and used RNAi-based protein depletion to extend the list of proteins shown to be required for APB formation to include PML, TRF1, TRF2, TIN2, RAP1, MRE11 and RAD50 [13]. It was also reported that the structural maintenance of chromosomes SMC5/6 complex localizes to APBs in ALT cells and sumoylates TRF1 and TRF2, and that this plays an essential role in APB formation [26].

Although definitive evidence is still lacking, it has long been thought that APBs might have an integral role in the ALT mechanism [11], [12], [19], [27], [28] and, consistent with this suggestion, inhibition of ALT in some somatic cell hybrids formed by fusion of ALT and telomerase-positive cell lines resulted in a substantial decrease in APBs [29]. Moreover, when ALT was inhibited by sequestration or depletion of the MRE11/RAD50/NBS1 homologous recombination complex, this was accompanied by suppression of APBs, providing further evidence for a direct link between APBs and ALT activity [25], [30]. However, large APBs are found in ∼5% of exponentially dividing (“normal”) ALT cells [11], and most of the APB-positive cells in these “normal” ALT populations did not incorporate BrdU within 24 hours (which exceeded their average doubling time), and also displayed an enlarged, flat morphology, indicating that they are most likely growth-arrested or senescent. This association with growth arrest/senescence appears paradoxical if APBs are actually involved in the ALT mechanism, and we have recently discussed the possibility that APBs are functionally heterogeneous, with only a subset being directly involved in ALT-mediated telomere lengthening [4].

Another possibility is that APBs are simply a byproduct of the ALT process, and this notion was supported by our recent finding that heterochromatin protein 1 (HP1), which is involved in chromatin compaction, is present in APBs, and that knockdown of HP1α and/or HP1γ significantly inhibited p53/p21-mediated APB induction[31]. This indicated that HP1 is required for APB formation, and that it is likely that APBs contain “closed” telomeric chromatin and are not the sites of telomere-telomere recombination. In an attempt to understand the nature of HP1-mediated chromatin compaction in APB formation, we have now investigated possible roles for two chromatin regulators/histone chaperones HIRA and ASF1a in the process of APB formation.

HIRA has features of both the yeast Hir1p and Hir2p proteins [32], while ASF1a and ASF1b are two orthologs of yeast Asf1p protein [33], [34]. Yeast Hir and Asf1p proteins contribute to formation of transcriptionally silent heterochromatin, which is largely responsible for silencing telomeres and mating loci [35]–[40]. Telomeric silencing in yeast requires physical interaction between Hir and Asf1p proteins [41]. Similarly, human HIRA and ASF1a display histone chaperone activity [42]–[44], and their physical association is essential for formation of senescence-associated heterochromatin foci (SAHF) [45], [46], which is one of the molecular characteristics of normal senescent cells [45], [47]. SAHF contain several molecular markers of transcriptionally silent heterochromatin such as HP1. It has been previously shown that, prior to their incorporation into SAHF, HP1 proteins are transiently localized to PML bodies, where they colocalize with HIRA [45]. More importantly, recruitment of HIRA into PML bodies is one of the earliest steps towards senescence in human cells [45]. Although its function inside PML bodies is largely unknown, blocking entry of HIRA to PML bodies prevents formation of SAHF [48].

We found that HIRA is present inside APBs in ALT cells in which the p53/p21 pathway has been restored and showed, for the first time, that ASF1a colocalizes with HIRA inside APBs in ALT cells as well as in PML bodies in normal cells. Knockdown of HIRA, but not ASF1a, significantly decreased the p53/p21-mediated induction of APBs, suggesting that HIRA is required for HP1-mediated APB formation in this context. Furthermore, we found that ASF1a accumulated inside nucleoli of proliferating but not senescent cells, demonstrating a link between senescence and release of ASF1a from nucleolar sequestration.

## Results

### HIRA and ASF1a are localized in APBs upon p53/p21-mediated senescence

As noted above, large APBs (henceforth referred to as APBs) are usually found in ∼5% of cells within asynchronously growing ALT cell populations [11]. They can be detected interchangeably by either telomeric FISH or immunostaining of TRF1 or TRF2 [13], [25], [31]. Because not every PML body in ALT cells contains telomeric components, the term “PML body” is not equivalent to the term “APB”; “APB” is used if telomere components are detected, otherwise “PML body” is used. In this study, APBs were generally detected by visualizing TRF1 or TRF2 within a PML body, but were also identified as large, bright TRF1, TRF2 or telomeric DNA foci (Figure S1), because the quantity of telomeric DNA and telomeric binding proteins that APBs contain is greater than the amount present at individual telomeres. As shown in Figure S1, sometimes individual telomeres can form small foci with PML, which are much fainter than APBs. Similar structures have recently been reported in human astrocytoma tissues [49].

The proportion of APB-positive cells can be greatly increased by restoration of the p53 pathway in p53-negative ALT cells, and this increase in APB formation is mediated by HP1 proteins [31]. On the basis of this information and the observation that HP1 proteins colocalize with HIRA in PML bodies upon onset of senescence in normal human fibroblasts [45], we wanted to determine whether HIRA and its binding partner ASF1a are involved in APB formation. First, we examined localization of HIRA and ASF1a in relation to APBs in two p53-estrogen receptor (ER) fusion gene-transfected IIICF/c cell lines, c/p53ER/7 and c/p53ER/8 (abbreviated to C7 and C8), in which p53 function can be activated by exposure to 4-hydroxytamoxifen (4OHT) [50]. IIICF/c is an ALT cell line [51] derived from IIICF Li-Fraumeni Syndrome fibroblasts containing one mutant (essentially null) and one wild-type (wt) TP53 allele [52], that became immortalized spontaneously via a series of genetic changes that included loss of the wt TP53 allele [51].

**Figure 1:**
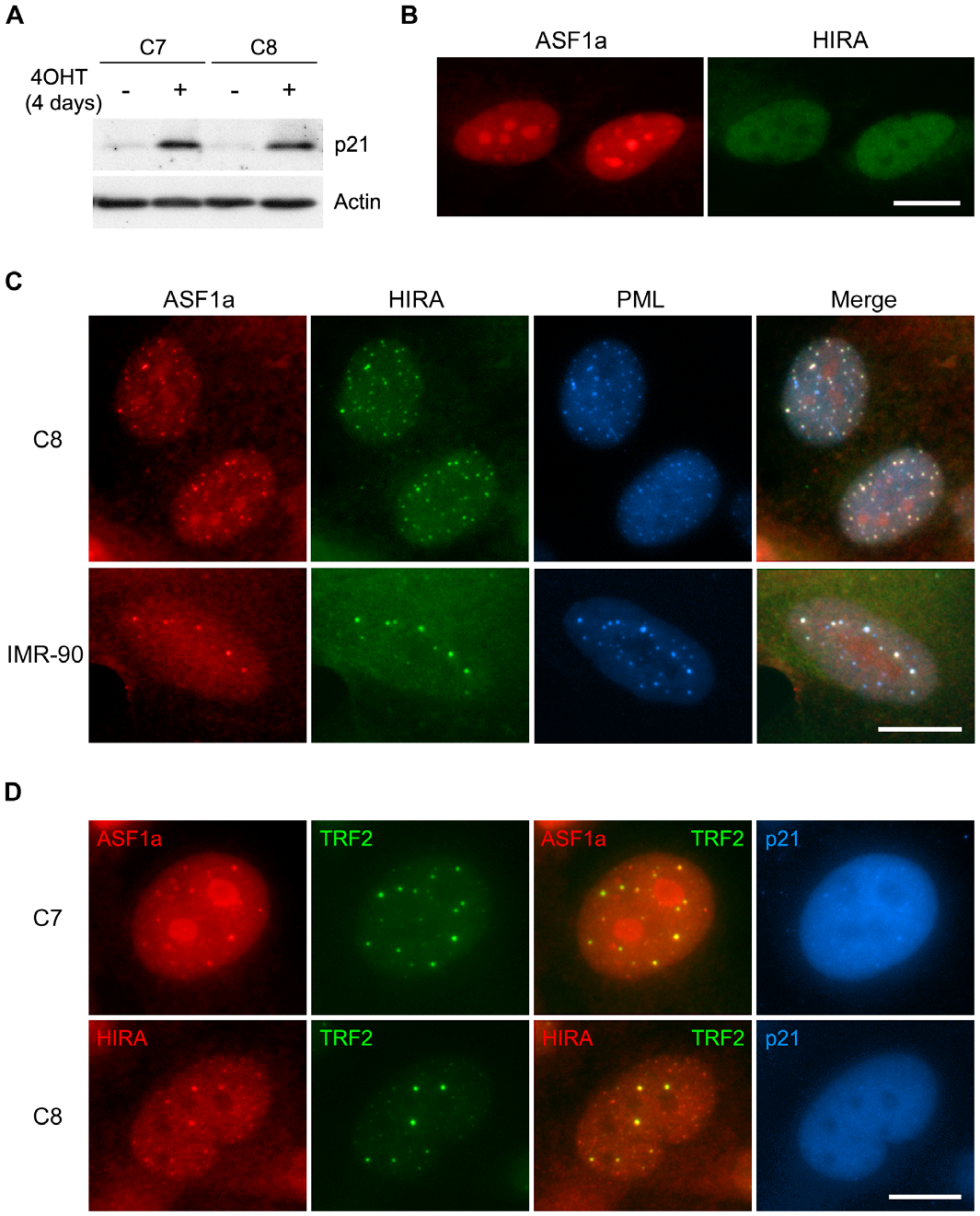
Presence of ASF1a and HIRA inside PML bodies in ALT cells and normal fibroblasts. (A) p21 was upregulated in C7 and C8 cells, that express a p53-ER fusion protein, after 4 days of 4OHT-treatment. The western blot was probed with the indicated antibodies. (B) Untreated, proliferating C7 cells were stained for ASF1a and HIRA. No ASF1a and HIRA foci were present in these nuclei. (C) Triple immunofluorescence of ASF1a, HIRA and PML in C8 cells treated with 4OHT for 4 days, and in senescent IMR-90 fibroblasts, showed colocalization of ASF1a and HIRA in PML bodies. (D) Immunostaining showed colocalization of ALT-associated PML bodies (APBs; visualized here as large TRF2 foci) and ASF1a or HIRA in C7 and C8 cells treated with 4OHT for 4 days. The antibodies used for this figure and subsequent figures are described in Materials and Methods and are only specified in the figure legends when Materials and Methods lists more than one antibody against that protein; here the mouse anti-HIRA (B, C), rabbit anti-HIRA (D), mouse anti-p21 (A), goat anti-p21 (D) and goat anti-PML (C) antibodies were used. Bars, 20 µm.

Treatment of C7 and C8 cells with 4OHT upregulated the level of p21 (Figure 1A), resulting in a senescent phenotype, and a significant increase in the proportion of cells containing APBs, some of which showing the presence of p21 inside APBs [31], within 4 days (Figure S2). Immunostaining analysis on asynchronously growing (untreated) C7 and C8 cells showed that HIRA and ASF1a were localized in the nucleus, with HIRA being excluded from and ASF1a being highly concentrated inside nucleoli (Figure 1B). In a small proportion (∼0.7%) of the cell population, there were nuclear foci outside of the nucleoli containing both HIRA and ASF1a. When C7 and C8 cells were treated with 4OHT for 4 days, there was a very large increase in the proportion of cells (∼24%) containing HIRA/ASF1a foci. In essentially all of these foci ASF1a was found to colocalize with HIRA inside PML bodies (Figure 1C). Triple immunostaining of TRF2, PML and HIRA or ASF1a on 4OHT-treated C7 and C8 cells revealed the presence of HIRA and ASF1a inside APBs in ∼55% of APB-positive C7 and C8 cells at day 4 of the treatment (Figure 1D). Some HIRA/ASF1a foci in these cells occurred in PML bodies other than APBs. HIRA and ASF1a were also found to colocalize inside PML bodies in >70% of the normal senescent IMR-90 fibroblast population (Figure 1C). This is the first time that ASF1a has been found to localize to PML bodies and colocalize with HIRA in senescent ALT or normal cells.

**Figure 2:**
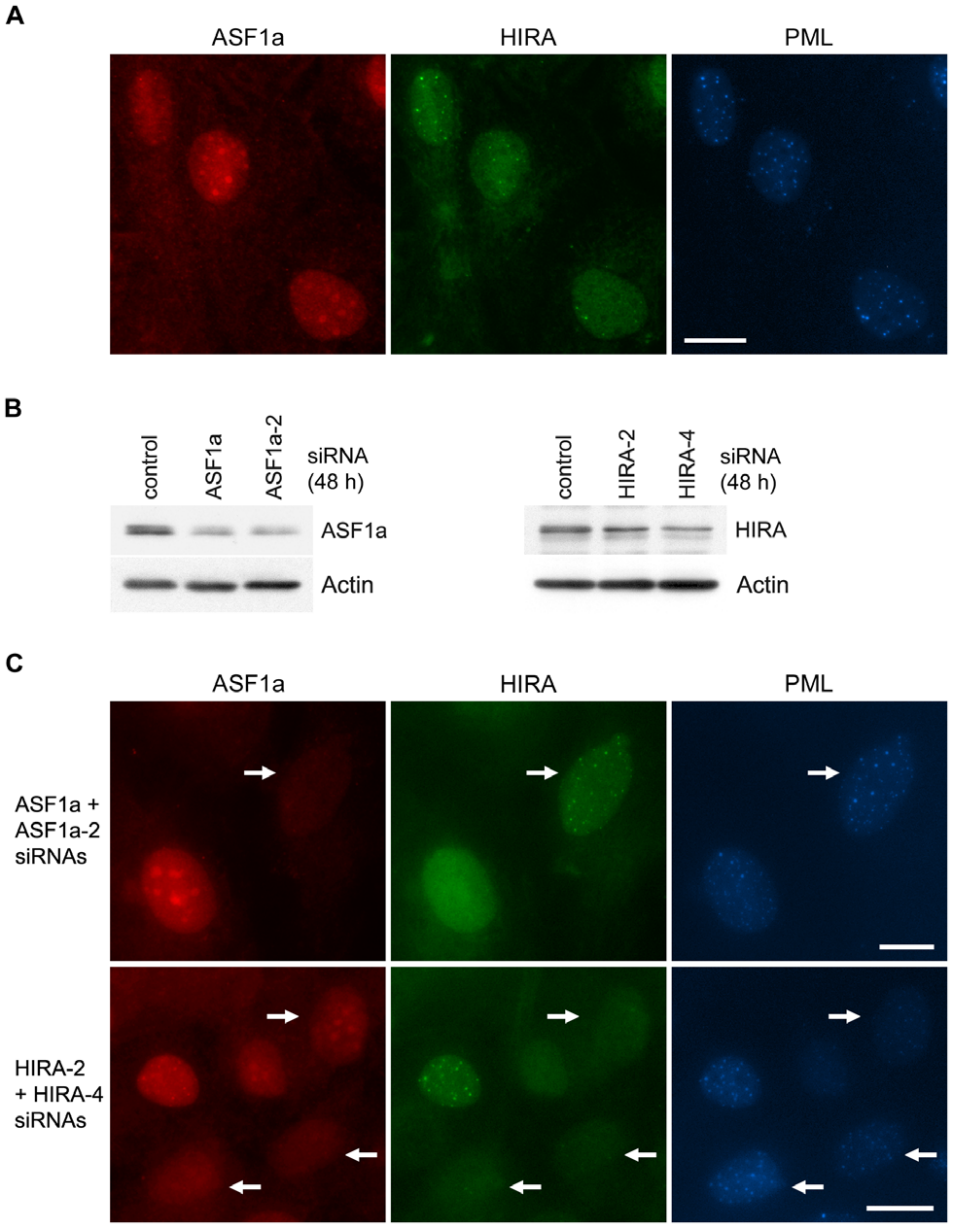
Localization of HIRA in PML bodies is independent of ASF1a. (A) C7 cells were triple stained for ASF1a, HIRA and PML 24 h after treatment with 4OHT. At this early time point, HIRA was more readily detected inside PML bodies than ASF1a. (B) The effectiveness of ASF1a siRNAs (ASF1a and ASF1a-2) and HIRA siRNAs (HIRA-2 and HIRA-4) was demonstrated in IIICF-T/B3 cells by Western blots, that were probed with the indicated antibodies. (C) Triple immunostaining of ASF1a, HIRA and PML in C7 cells after treatment with the indicated combinations of siRNAs for 5 days, and with 4OHT for 3 days before the end of siRNA-treatment. Distinct HIRA foci colocalized with PML bodies in cells depleted of ASF1a, while ASF1a was absent from PML bodies in cells depleted of HIRA. The arrows indicate cells with knockdown of ASF1a (top row) or HIRA (bottom row). Antibodies used here included mouse anti-HIRA (A, C), rabbit anti-HIRA (B) and goat anti-PML (A, C). Bars, 20 µm.

Distinctive HIRA foci were able to be detected inside PML bodies as early as one day after 4OHT-treatment, at which point the ASF1a colocalization was weak or non-existent (Figure 2A), in contrast to the strong ASF1a foci at day 4 (Figure 1C). These data suggest that HIRA is recruited to PML bodies earlier than ASF1a, implying that localization of HIRA to PML bodies is not dependent on its physical association with ASF1a. To confirm this, we performed knockdown of HIRA or ASF1a, 48 h prior to the onset of 4OHT-mediated induction of p53, with two HIRA siRNAs (HIRA-2 and HIRA-4) or two ASF1a siRNAs (ASF1a and ASF1a-2), the effectiveness of which was demonstrated by Western analysis (Figure 2B). We found that HIRA was present inside the PML bodies in the cells depleted of ASF1a, but not vice versa for ASF1a in HIRA-depleted cells (Figure 2C). These data indicate that HIRA is able to translocate to PML bodies independent of ASF1a.

### HIRA, but not ASF1a, is required for p53/p21-mediated APB induction

**Figure 3:**
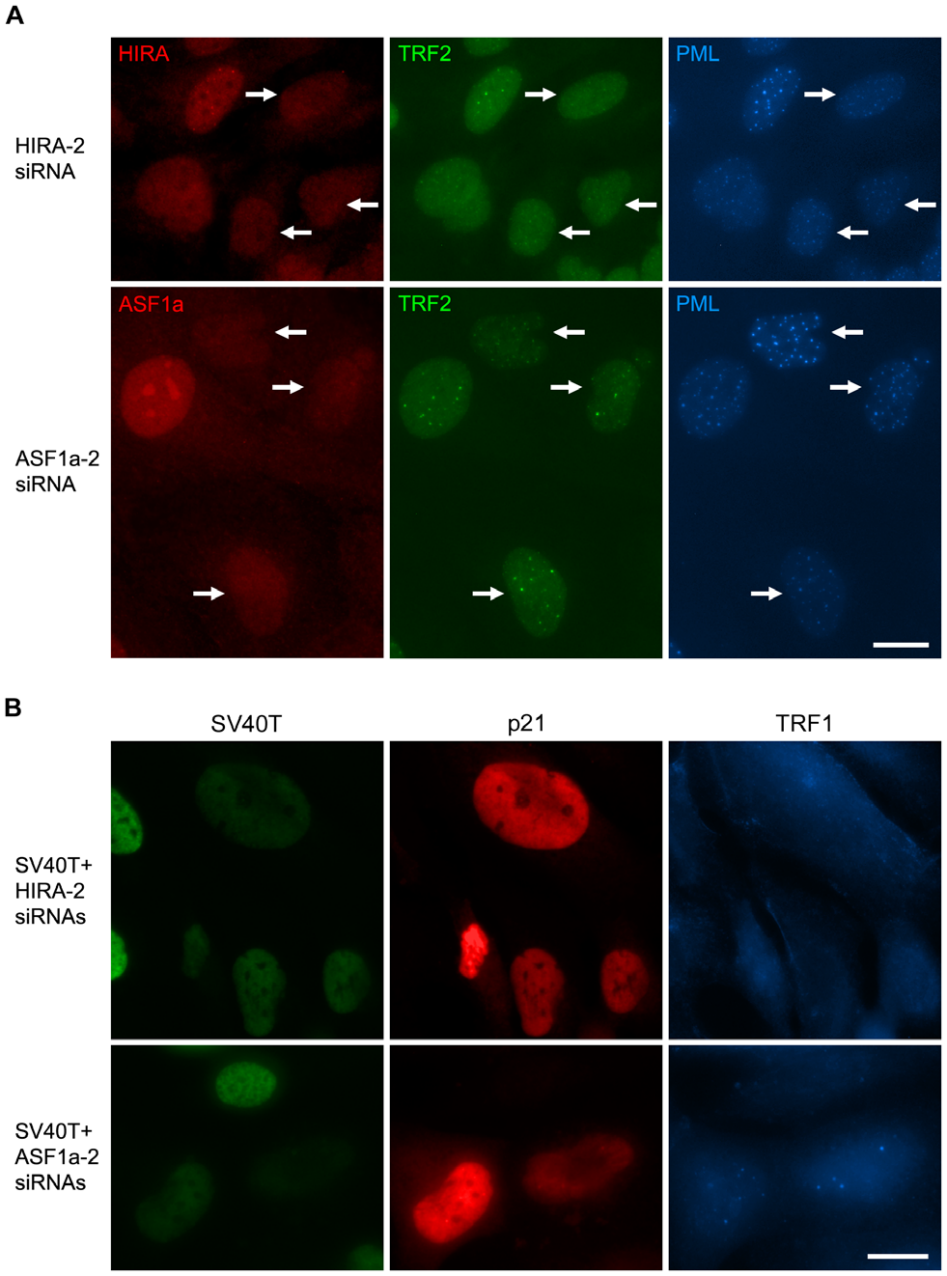
HIRA is required for p53/p21-mediated induction of APBs. (A) Triple immunostaining with the indicated antibodies in C7 cells after treatment with HIRA or ASF1a siRNA for 5 days, and with 4OHT for 3 days before the end of siRNA-treatment. Knockdown of HIRA, but not ASF1a, largely prevented p53/p21-mediated induction of APBs. The arrows indicate cells with knockdown of HIRA (top row) or ASF1a (bottom row). (B) IIICF-T/B3 cells were stained for SV40T, p21 and TRF1 4 days after transfection of the indicated combinations of siRNAs. HIRA siRNA (HIRA-2), but not ASF1a siRNA (ASF1a-2), significantly suppressed SV40T siRNA-mediated induction of APBs (visualized here as large TRF1 foci). Antibodies used here included rabbit anti-HIRA (A), goat anti-p21 (B) and goat anti-PML (A). Bars, 20 µm.

The presence of both HIRA and ASF1a inside APBs led us to determine whether HIRA and/or ASF1a are involved in p53/p21-mediated induction of APBs. HIRA or ASF1a siRNAs were transfected into C7 cells 48 h prior to a 3-day treatment with 4OHT. We found that knockdown of HIRA significantly inhibited the p53/p21-mediated induction of APBs, whereas knockdown of ASF1a had no significant impact on APB formation (Figure 3A). However, when quantitation was carried out for the total cell populations, only a ∼20% reduction in APB formation was obtained for cells treated with HIRA-siRNAs (Table 1). The reasons for this apparent discrepancy are that (1) only a portion of the siRNA-treated cell population was effectively depleted of the target protein due in part to transfection efficiency, and in part to varied efficacies of individual siRNAs; and (2) only 50–60% of the C7 or C8 cell populations are p53/p21-inducible upon addition of 4OHT. These two factors limit the number of cells which are both p53/p21-induced and also depleted of the target protein.

**Table 1:** Proportion of APB-positive C7 cells after treatment with siRNAs and 4-hydroxytamoxifen (4OHT).

siRNA-treatment^a^	Total	APB+(%)
none	216	92 (42.6)
control	203	79 (38.9)
ASF1a	207	84 (40.6)
ASF1a-2	206	90 (43.7)
HIRA-2	211	67 (31.8)
HIRA-4	218	61 (28.0)

a Cells were transfected with 10 nM siRNA 48 h prior to the addition of 1 µM 4OHT, which was maintained for another 3 days. None, no siRNA-treatment; control, non-silencing control siRNA.

To overcome this intrinsic property of the p53ER inducible system, we used another ALT cell line, IIICF-T/B3, that was established by transfecting IIICF cells with an SV40 early region expression plasmid [53]; these cells retain a wt TP53 allele. As shown previously [31], p53 function can be restored in IIICF-T/B3 cells by knockdown of SV40 large T antigen (LTAg) with an siRNA against the SV40 early region transcripts (SV40T siRNA), which results in upregulation of p21, a senescent phenotype, and a significant increase in APB formation (Figure S3). Simultaneous knockdown of LTAg and any target proteins in IIICF-T/B3 cells can achieve, to a large extent, both p53/p21-induction and depletion of the target proteins. Thus, counting only the cells depleted of LTAg should result in much more accurate quantitation. We therefore performed double knockdown of LTAg and HIRA or ASF1a in IIICF-T/B3 cells with a combination of SV40T siRNA and HIRA siRNAs (HIRA-2 or HIRA-4), or ASF1a siRNAs (ASF1a or ASF1a-2). APB-positivity was scored for the LTAg-depleted cells, revealing that knockdown of HIRA inhibited the p53/p21-mediated induction of APBs by ∼40%, whereas ASF1a had no apparent effects on APB induction (Figure 3B and Table 2). Moreover, simultaneous knockdown of HIRA and ASF1a had no additive effect in inhibiting APB formation as compared to knockdown of HIRA alone (Table 3), which confirms the conclusion that ASF1a has no involvement in APB formation.

**Table 2:** Proportion of APB-positive IIICF-T/B3 cells after siRNA-treatment.

siRNA-treatment^a^	Total (SV40T-)^b^	APB+(%)
SV40T+C	104	57 (54.8)
SV40T+p21-6	110	28 (25.5)
SV40T+ASF1a	112	63 (56.3)
SV40T+ASF1a-2	109	57 (52.3)
SV40T+HIRA-2	107	38 (35.5)
SV40T+HIRA-4	106	34 (32.1)
SV40T+C	108	66 (61.1)
SV40T+p21-6	103	29 (28.2)
SV40T+ASF1a	99	58 (58.6)
SV40T+ASF1a-2	115	62 (53.9)
SV40T+HIRA-2	104	35 (33.7)
SV40T+HIRA-4	110	40 (36.4)

a Cells were treated with 10 nM siRNA per target for 4 days before being fixed for immunostaining. C, non-silencing control siRNA.

b Only cells that were negative by immunostaining for SV40T were examined for APBs.

**Table 3:** Proportion of APB-positive IIICF-T/B3 cells after siRNA-treatment.

siRNA-treatment^a^	Total (SV40T-)^b^	APB+(%)
SV40T+C+C	103	60 (58.3)
SV40T+ASF1a-2+ASF1a-2	108	65 (60.2)
SV40T+ASF1a-2+HIRA-4	102	36 (35.3)
SV40T+HIRA-4+HIRA-4	111	33 (29.7)
SV40T+HP1α-2+HP1γ-6	107	28 (26.2)
SV40T+C+C	123	69 (56.1)
SV40T+C+HIRA-4	115	40 (34.8)
SV40T+ASF1a-2+HIRA-4	126	42 (33.3)
SV40T+C+C	110	63 (57.3)
SV40T+C+p21-6	113	34 (30.1)
SV40T+C+ASF1b	107	59 (55.1)
SV40T+C+ASF1b-2	105	62 (59.0)
SV40T+ASF1a+ASF1b	114	66 (57.9)
SV40T+ASF1a-2+ASF1b-2	115	71 (61.7)

a Cells were treated with 10 nM siRNA per target for 4 days before being fixed for immunostaining. C, non-silencing control siRNA.

b Only cells that were negative by immunostaining for SV40T were examined for APBs.

**Figure 4:**
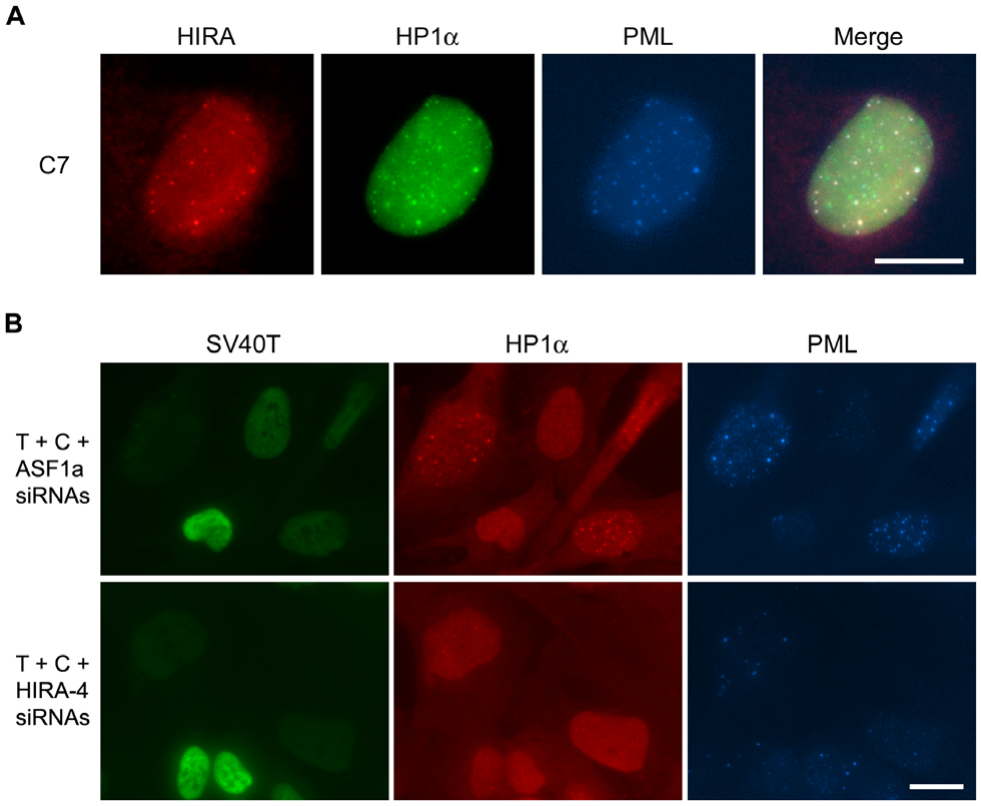
HIRA is required for p53/p21-mediated formation of large HP1 foci. (A) Triple immunostaining showed colocalization between HIRA and HP1α inside PML bodies in C7 cells treated with 4OHT for 2 days. (B) IIICF-T/B3 cells were stained for SV40T, HP1α and PML 4 days after transfection of the indicated combinations of siRNAs (T, SV40T; C, control). HIRA siRNA (HIRA-4), but not ASF1a siRNA (ASF1a), largely blocked SV40T siRNA-mediated induction of large HP1α foci. Antibodies used here included rabbit anti-HIRA (A), mouse anti-HP1α (A), rabbit anti-HP1α (B) and goat anti-PML (A, B). Bars, 20 µm.

HP1 proteins are present in APBs and are required for APB formation upon p53/p21-mediated growth arrest/senescence [31]. As expected, HIRA colocalized with HP1 inside PML bodies in 4OHT-treated C7 and C8 cells (Figure 4A, and data not shown). To examine possible involvement of HIRA in the formation of APB-associated, large HP1α foci, we transfected IIICF-T/B3 cells with a combination of SV40T siRNA and siRNAs against HIRA, ASF1a or HP1α. We found that knockdown of HIRA reduced p53/p21-mediated induction of large HP1α foci by 37–45%, whereas depletion of ASF1a resulted in no apparent reduction (Figure 4B and Table 4). Although a greater reduction was achieved by the control HP1α-knockdown, the amount of reduction obtained by HIRA-knockdown was significant, and compatible with the extent to which it reduced APB formation, further supporting the conclusion that HIRA is indispensable for p53/p21-mediated induction of APBs.

**Table 4:** Proportion of IIICF-T/B3 cells with large HP1α-foci after siRNA-treatment.

siRNA-treatment^a^	Total (SV40T-)^b^	Large HP1α foci+ (%)
SV40T+C+C	116	72 (62.1)
SV40T+C+HP1α-2	127	20 (15.7)
SV40T+C+ASF1a	118	75 (63.6)
SV40T+C+ASF1a-2	114	67 (58.8)
SV40T+C+HIRA-2	129	44 (34.1)
SV40T+C+HIRA-4	123	47 (38.2)
SV40T+C+C	105	71 (67.6)
SV40T+C+HIRA-4	112	48 (42.9)
SV40T+HIRA-4+HIRA-4	110	37 (33.6)
SV40T+HIRA-4+ASF1a-2	105	42 (40.0)

a Cells were treated with 10 nM siRNA per target for 4 days before being fixed for immunostaining. C, non-silencing control siRNA.

b Only cells that were negative by immunostaining for SV40T were examined for large HP1α-foci.

Although ASF1a, unlike HIRA, is dispensable for p53/p21-mediated APB formation, the occurrence of APBs in senescent ALT cells made it important to determine whether either or both of these proteins are required for SAHF formation in 4OHT-treated C7 and C8 cells. We therefore performed knockdown of ASF1a or HIRA in C7 and C8 cells, 48 h prior to a 4-day treatment with 4OHT, with ASF1a or HIRA siRNAs, and found that depletion of ASF1a or HIRA, resulted in a significant reduction on SAHF formation (Figure S4 and Table S1), which is consistent with the previous studies indicating that both proteins are required for SAHF formation in normal senescent fibroblasts [45], [48].

### ASF1b is absent from APBs and dispensable for APB formation

**Figure 5:**
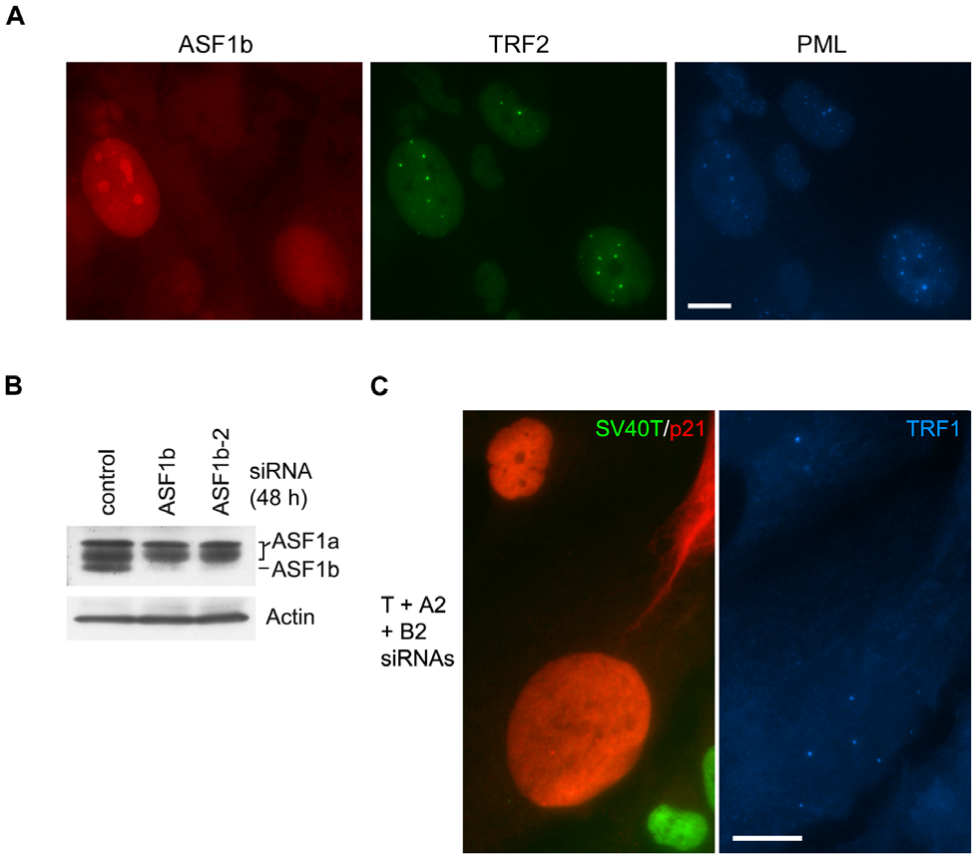
ASF1a and ASF1b are both dispensable for APB formation. (A) Triple immunostaining of ASF1b, TRF2 and PML in C7 cells treated with 4OHT for 4 days. No ASF1b foci were observed inside APBs. (B) The effectiveness of ASF1b siRNAs (ASF1b and ASF1b-2) was demonstrated in IIICF-T/B3 cells by immunoblotting. The blots were probed with the indicated antibodies. (C) Triple staining of SV40T, p21 and TRF1 in IIICF-T/B3 cells treated with the indicated combination of siRNAs (T, SV40T; A2, ASF1a-2; B2, ASF1b-2) for 4 days. APBs (visualized here as large TRF1 foci) were still detected in cells that were negative for SV40T. Antibodies used here included rabbit mAb anti-ASF1b (A), rabbit anti-ASF1b (B), goat anti-p21 (C) and goat anti-PML (A). Bars, 20 µm.

Human cells have two ASF1 paralogs, ASF1a and ASF1b. Unlike ASF1a, ASF1b does not physically intact with HIRA [43], [45], but instead forms complexes with chromatin assembly factor 1 (CAF-1) [54]. Despite the lack of physical interaction between ASF1b and HIRA, it was of interest to determine whether there is any functional redundancy between ASF1a and ASF1b with regard to p53/p21-mediated APB formation. We first examined the localization of ASF1b by triple immunostaining of ASF1b, TRF2 and PML, and found that ASF1b did not form foci inside APBs, but was distributed homogeneously in the nuclei (Figure 5A). We then performed triple knockdown of LTAg, ASF1a and ASF1b in IIICF-T/B3 cells with SV40T siRNA, ASF1a siRNAs, and two siRNAs against ASF1b (ASF1b and ASF1b-2), the effectiveness of which was demonstrated by Western analysis (Figure 5B). We found that, like knockdown of ASF1a and ASF1b individually, simultaneous knockdown of these proteins did not block p53/p21-mediated APB induction (Figure 5C and Table 3). The data indicate that neither ASF1a nor ASF1b is required for APB formation and that they have no functional redundancy in this context.

### Strong nucleolar presence of ASF1a in proliferating cancer and normal cells

**Figure 6:**
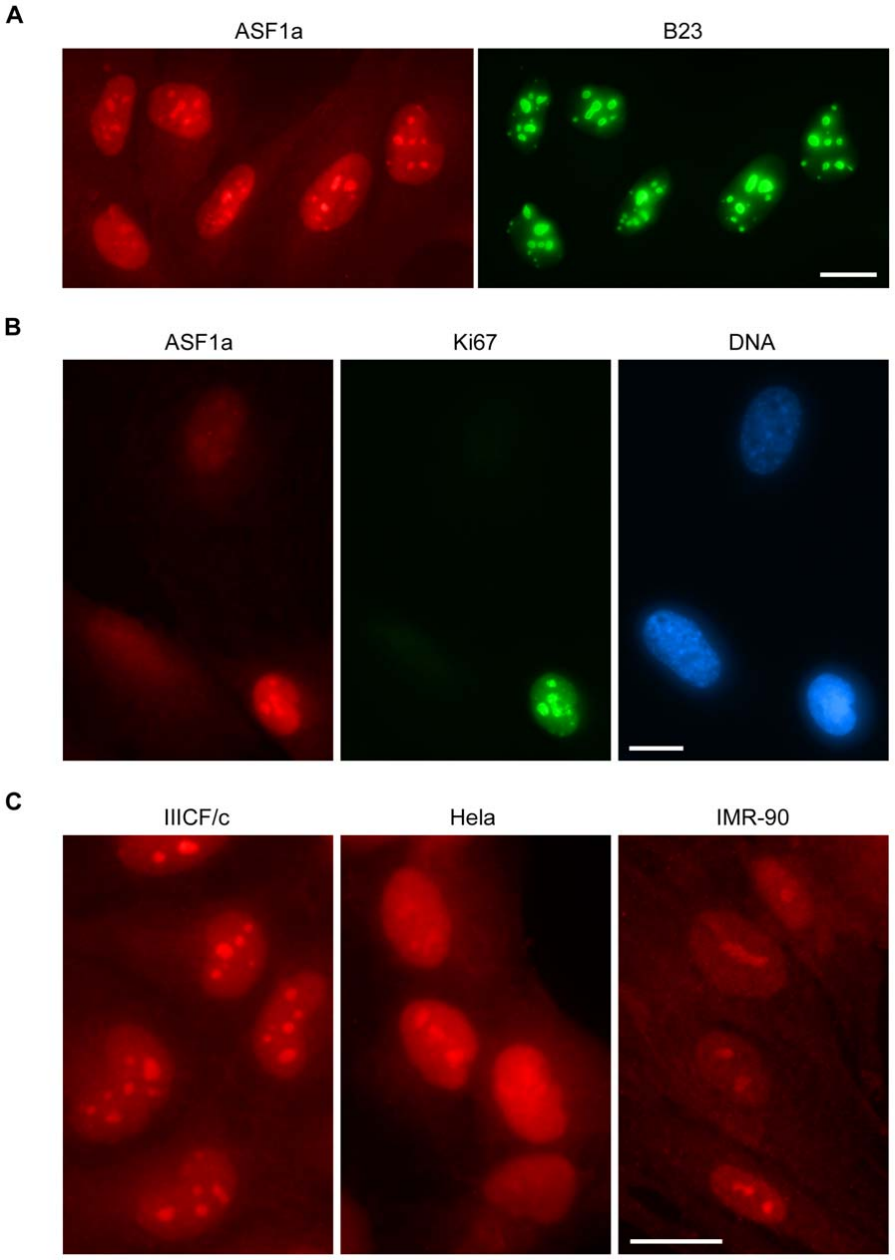
Accumulation of ASF1a inside nucleoli. (A) Immunostaining of proliferating C7 cells showed colocalization between ASF1a and the nucleolar marker B23. (B) Triple staining of ASF1a, Ki67 and DNA in C7 cells treated with 4OHT for 4 days. ASF1a was largely absent from the nucleoli of Ki67-negative cells. (C) ASF1a-staining of proliferating IIICF/c, Hela and IMR-90 cells showed nucleolar localization of ASF1a in all three cell lines. Bars, 20 µm.

During the course of the study, we found that ASF1a accumulated inside the nucleoli of proliferating C7 and C8 cells (Figure 6A), and upon 4OHT-treatment, this accumulation gradually disappeared from the majority of cells in the population (Figure 6B), suggesting that nucleolar localization of ASF1a is associated with cell growth and its release from nucleoli is associated with growth arrest. The specificity of the nucleolar ASF1a staining was confirmed by two ASF1a siRNAs (Figure S5, and data not shown), and nucleolar markers B23 (nucleophosmin/NPM) and ki-67 (Figure 6A and 6B). We extended the analysis of ASF1a localization to other cell types. Nucleolar ASF1a accumulation was observed in IIICF/c cells, normal IMR-90 fibroblasts and telomerase-positive HeLa cells (Figure 6C). HIRA did not accumulate inside the nucleolus (Figure 1B), and double staining of ASF1b and B23 in C7 and C8 cells revealed that only a small portion of the population displayed prominent nucleolar ASF1b localization, which is much less frequent than that of ASF1a (Figure S6), suggesting that ASF1a and ASF1b may have different roles in regulating cell growth.

## Discussion

We previously showed that the heterochromatin proteins, HP1α and γ, are required for formation of APBs in ALT cells upon p53/p21-mediated senescence [31]. Here we have shown that another heterochromatin regulator, HIRA, is involved in both APB formation and SAHF formation, a key step in establishing the senescent phenotype, and a further heterochromatin regulator, ASF1a, is involved in SAHF formation. These findings tighten the correlation between induction of APBs and senescence, consistent with the observation that knockdown of p21, a major downstream regulator of p53-mediated senescence, significantly reduces p53-mediated induction of APBs [31], and provide new insights into the mechanisms involved in this process.

We have not found any fundamental differences between APBs in asynchronously growing ALT cell populations and those where senescence is experimentally induced. Only a small minority of asynchronously growing ALT cells have large APBs, and most of these appear to be growth arrested/senescent (T. Yeager and R. Reddel, unpublished data); the causes of growth arrest in these cells may include mitotic accidents. This is consistent with the results from our recent study [31] showing that APB formation is associated with growth arrest/senescence. The demonstration of HIRA's involvement in HP1-mediated APB formation further supports the notion that APBs are likely to contain “closed” telomeric chromatin, which is unlikely to permit telomere-telomere recombination. Given this and the lack of direct evidence for de-novo telomere lengthening inside APBs, we suggest that APBs may simply be a byproduct of the ALT process.

Although the function of HIRA inside PML bodies is largely unknown, its relocalization there is an early step in the senescence program induced by short telomeres, activated oncogenes or cell stress [48], and physical interaction of HIRA and ASF1a is required for SAHF formation in senescing normal cells [45]. It has been proposed that HIRA-containing complexes are assembled or modified in PML bodies, and then form complexes with ASF1a and histone H3 to catalyze formation of SAHF elsewhere in the nucleus [55]. We demonstrated here for the first time that ASF1a colocalizes with HIRA in PML bodies of senescing fibroblasts (and in APBs in ALT cells), suggesting that PML bodies are the location for the HIRA/ASF1a interaction. Intriguingly, we found that ASF1a is sequestered in the nucleoli of proliferating cells and is released when senescence is induced, presumably permitting its interaction with HIRA in PML bodies. This is consistent with a growing body of evidence that the nucleolus has a role in regulating specific aspects of cell cycle progression via protein modifications and protein sequestration [56].

HIRA has been shown to be present at telomeres where it is thought to function as a histone chaperone [57]. A recent study [58] has shown that ubinuclein 1 (UBN1) colocalizes with HIRA inside PML bodies in senescent cells. UBN1 is not only a component of HIRA/ASF1a complexes, but is also associated with histone methyltransferase (HMTase) activity toward lysine 9 of histone H3 (H3K9). HP1 proteins are usually recruited to chromatin, including telomeric chromatin, through their affinity for trimethylated H3K9 residues [59], [60]. Consistent with these observations, we found that reducing APB formation by knockdown of HIRA was accompanied by a similar reduction in formation of large HP1 foci, suggesting that HIRA is required for the association of HP1 with telomeric chromatin inside PML bodies to form APBs. Based on these data, we speculate that HIRA may, through its association with UBN1 and HMTase, modify the methylation state of histone H3 bound to telomeric DNA as a prerequisite for HP1-mediated compaction of this DNA and APB formation.

HIRA formed foci inside PML bodies earlier than ASF1a in ALT cells following restoration of the p53/p21 pathway, and was able to do so in ASF1a-depleted cells, and knockdown of ASF1a had no significant effects on formation of APBs and large HP1 foci. These data are consistent with previous reports that translocation of HIRA to PML bodies in normal pre-senescent fibroblasts is independent of ASF1a [48], [61]. In addition to the observation that HIRA and ASF1a are both required for SAHF formation in senescing ALT cells (this study), other relevant observations are that interaction between HIRA and ASF1a is required for formation of SAHF in normal senescent fibroblasts, formation of SAHF occurs much later than recruitment of HIRA into PML bodies upon senescence, formation of SAHF is not catalyzed inside PML bodies, and telomeres are excluded from SAHF [45], [55], [62]. It is therefore plausible that HIRA could play an ASF1a-independent role via its association with UBN1 and HMTase after its entry to PML bodies, and then interact with ASF1a to form complexes before leaving PML bodies to catalyze formation of SAHF elsewhere in the nucleus.

We found no ASF1b foci inside APBs or PML bodies and no evidence that ASF1b had a functional role in APB formation, in agreement with the observation that ASF1b is unable to bind HIRA [43], [45]. ASF1b had much less nucleolar localization than ASF1a. These two isoforms appear to have distinct roles, e.g., ASF1a is involved in both DNA synthesis-coupled and -independent nucleosome assembly, and ASF1b is only involved in the former [43]–[45], [54].

**Figure 7:**
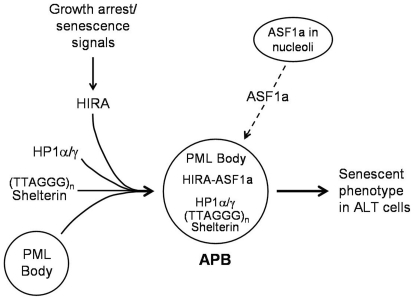
A hypothetical model for HIRA and ASF1a in APB formation and senescence in ALT cells. Growth arrest/senescence signals trigger relocalization of HIRA to PML bodies, which leads to the subsequent translocation of HP1α/γ, telomeric DNA and its associated shelterin proteins, each of which is essential for APB formation. The dashed line indicates that translocation of ASF1a to PML bodies and its colocalization there with HIRA is not essential for APB formation, even though HIRA and ASF1a are both required for the establishment of the senescent phenotype in ALT cells.

In summary, the results from our study show that HIRA but not ASF1a is required for p53/p21-mediated induction of APBs, suggesting that HIRA plays a unique, ASF1a-independent role in this process, and that PML bodies in ALT cells could serve as sites that integrate two senescence-related cellular processes: (1) compaction of telomeric DNA by HIRA and HP1, and (2) assembly of the HIRA/ASF1a complexes needed for SAHF formation (Figure 7). The requirement for HIRA in both APB and SAHF formation supports the close correlation between APBs and senescence of cells that use the ALT mechanism.

## Materials and Methods

### Cell culture

The spontaneously immortalized Li-Fraumeni Syndrome fibroblast line IIICF/c [51], human cervical carcinoma cell line HeLa and normal human fibroblast strain IMR-90 (ATCC, Manassas, VA, USA) were cultured in Dulbecco's modified Eagle's medium (DMEM; GIBCO, Rockville, MD, USA). The p53-estrogen receptor (ER) fusion gene-transfected IIICF/c cell lines, c/p53ER/7 (C7) and c/p53ER/8 (C8), were cultured in phenol red-free DMEM (GIBCO) containing 1 µg/ml puromycin [50]. SV40-immortalized Li-Fraumeni Syndrome fibroblast cell line IIICF-T/B3 [53] was cultured in RPMI 1640 medium (GIBCO). All culture media contained 10% fetal bovine serum (FBS) and 50 µg/ml gentamicin, and cultures were incubated in a 5% CO_2_ humidified atmosphere at 37°C.

For p53-induction experiments, C7 and C8 cells were seeded in phenol red-free DMEM and grown to 30–40% confluency. The cell cultures were treated with 1 µM 4OHT or 0.01% ethanol as vehicle control. After various time periods, cells were either fixed for immunostaining, or harvested for isolation of protein.

### Antibodies

The following antibodies were used in this study: rabbit anti-ASF1a and mouse anti-B23 (ProteinTech Group, Chicago, IL, USA); rabbit anti-ASF1b (mAb, C70E2), anti-ASF1b and anti-HP1α (Cell Signaling Technology, Boston, MA, USA); rabbit anti-HIRA (Abgent, San Diego, CA, USA); mouse anti-HIRA (Abnova, Taipei, Taiwan); mouse anti-p21, anti-TRF2 and anti-Ki67 (BD Biosciences, San Jose, CA, USA); goat anti-p21 (R&D Systems, Minneapolis, MN, USA); mouse anti-p53 (DO1), goat anti-PML (N-19) and mouse anti-PML (Santa Cruz Biotechnology, Santa Cruz, CA, USA); mouse anti-TRF2 and anti-HP1α (Upstate Biotechnology, Waltham, MA, USA). Mouse anti-SV40T (PAb108) was purified from the supernatant of hybridoma TIB-230 (ATCC), and polyclonal anti-TRF1 rabbit serum was raised against a TRF1 peptide, residues 13–35.

### RNA interference

The following siRNAs were designed and synthesized by Qiagen (Hilden, Germany): for p21, 5′-CAGTTTGTGTGTCTTAATTAT-3′ (p21-6) and for HP1γ, 5′-CTGGTTACTTTGAACAAATAA-3′ (HP1γ-6). The following siRNAs were synthesized by Qiagen: for SV40T, 5′-AAAATTGTGTACCTTTAGCTT-3′ [63]; for HP1α (HP1α-2), 5′-GAGGAGCACAATACTTGGGAA-3′ [64]; for HIRA (HIRA-2 and HIRA-4), 5′-GGGGAGATGACAAACTGATTA-3′ and 5′-CAGAAATTCTAGCTACTCTGA-3′ [65]; for ASF1a (ASF1a and ASF1a-2), 5′- AAGTGAAGAATACGATCAAGT-3′ [66] and 5′-CTGAGACAGAATTAAGGGAAA-3′ [67]; and for ASF1b (ASF1b and ASF1b-2), 5′-AACAACGAGTACCTCAACCCT-3′ [66] and 5′-GGCGGACGACCTGGAGTGGAA-3′ [67]. The non-silencing control siRNA was purchased from Qiagen.

To determine the extent of knock-down, cells were transfected with 10 nM siRNA per target gene using HiPerFect transfection reagent (Qiagen) according to the manufacturer's instructions. For Western analysis, cells were seeded into six-well plates 1–2 days prior to siRNA transfection. After transfection for 48 h, cells were harvested for protein isolation.

### APB screening in SV40-immortalized cell lines by RNA interference

Cells were seeded into four-well chamber slides (Nunc, Naperville, IL, USA) two days before transfection of siRNAs. For double or triple knockdown experiments, 10 nM siRNA per target gene, and 10 nM SV40T siRNA, were transfected into cells using HiPerFect. Four days later, cells were fixed and immunostained for SV40T, p21 and TRF1 (large foci of which are recognized as APBs). Finally, APB-positivity was scored for the cells in which SV40T was depleted.

### Immunostaining, telomere FISH and fluorescence microscopy

Cells grown in four-well chamber slides were fixed for 15 min in 2% paraformaldehyde at room temperature, and then permeated with 0.5% Triton X-100 (Sigma-Aldrich, St Louis, MO, USA) at room temperature for 10 min or methanol:acetone (1∶1) at −20°C for 15 min. Cells were incubated overnight with primary antibodies at 4°C, and then were incubated with fluorescently conjugated secondary antibodies at room temperature for 40 min. In some cases, DAPI (Sigma-Aldrich) was included in the secondary incubation to visualize DNA. For detection of SAHF, the samples were stained with 0.13 µg/ml DAPI at room temperature for 2 min. Finally, the preparations were mounted in anti-fading medium containing DABCO (Sigma-Aldrich) or medium consisting of glycerol∶PBS (70%∶30%). The secondary antibodies used were as follows: FITC- or Texas Red-conjugated goat anti-mouse, FITC- or Texas Red-conjugated goat anti-rabbit, AMCA-, FITC- or Texas Red-conjugated donkey anti-mouse, AMCA-, FITC- or Texas Red-conjugated donkey anti-rabbit, AMCA- or Texas Red-conjugated donkey anti-goat (Jackson ImmunoResearch, West Grove, PA, USA).

Double staining of telomeric DNA and APB-associated proteins was performed as previously described [68]. Briefly, slides were first immunostained with primary and secondary antibodies, and then cross-linked with 4% formaldehyde and dehydrated. Telomere fluorescence in situ hybridization (FISH) was done by using a Cy3-conjugated telomere-specific peptide nucleic acid (PNA) probe (Applied Biosystems, Framingham, MA, USA).

The samples were examined on a Leica DMLB fluorescence microscope (Leica, Wetzlar, Germany). A PL-FLUOTAR 40x/NA 0.7 objective was used in this study. Images were recorded using a Spot cooled CCD camera (SPOT2; Diagnostic Instruments, Sterling Heights, MI, USA) and analyzed with PhotoShop 7.0 (Adobe, San Jose, CA, USA).

### SA-β-gal activity assay

Cells were grown in four-well chamber slides to 30–40% confluency and then treated with 1 µM 4OHT or 10 nM SV40T siRNA for 3 or 4 days. The SA-β-gal staining was performed with a SA-β-gal staining kit (Cell Signaling Technology) according to the manufacturer's instructions. The samples were examined on an Olympus IMT-2 inverted microscope (Olympus, Tokyo, Japan) with an A10PL 10x/NA 0.25 objective. Images were recorded using a digital camera (DP12; Olympus) and analyzed with PhotoShop 7.0 (Adobe).

### Immunoblotting

For immunoblotting analyses, cell lysates were prepared, electrophoretically separated on SDS-PAGE gels and electrotransferred to a nylon membrane as described previously [69]. Immunoblotting procedures were as recommended by the antibody suppliers. Horseradish peroxidase (HRP) conjugated goat anti-mouse, goat anti-rabbit or swine anti-rabbit IgG (DAKO, Glostrup, Denmark) were used as secondary antibodies.

## Supporting Information

Figure S1 APBs can be detected interchangeably by either telomeric FISH or immunostaining of TRF1 or TRF2. After 4 days of 4OHT-treatment, APBs were induced in C7 and C8 cells, where colocalization was observed between prominent TRF1 or TRF2 foci and large PML bodies (top row), and between telomeric DNA and TRF1 foci (bottom row). The antibodies used for this supplementary figure and the subsequent figures are described in Materials and Methods and are only specified in the figure legends when Materials and Methods lists more than one antibody against that protein; here the goat anti-PML antibody was used. Bar, 20 µm.
(TIF)

Figure S2 Induction of APBs is associated with p53/p21-mediated senescence. (A) SA-β-gal staining of C8 cells treated with 4OHT or ethanol vehicle for 4 days. SA-β-gal expression was found in 4OHT-treated C8 cells. (B) Double immunostaining of TRF1 and p21 in C8 cells treated with 4OHT or ethanol vehicle for 4 days. Large TRF1 foci were induced in cells positive for p21. Antibodies used here included mouse anti-p21 (B). Bars, 100 µm (A) and 20 µm (B).
(TIF)

Figure S3 Induction of APBs in SV40-immortalized ALT cells by siRNA-mediated knockdown of LTAg. (A) Western blots showed induction of p21 in IIICF-T/B3 cells 2 days after transfection of SV40T and control (T+C) siRNAs. This induction of p21 was effectively abrogated by p21 siRNA (p21-6). The blots were probed with the indicated antibodies. (B) SA-β-gal staining of IIICF-T/B3 cells treated with the indicated combinations of siRNAs for 3 days. Strong SA-β-gal expression was found in cells treated with T+C siRNAs. (C) IIICF-T/B3 cells were triple stained for SV40T, p21 and TRF1 4 days after SV40T siRNA transfection. APBs (visualized here as large TRF1 foci) were observed in cells with high levels of p21. Antibodies used here included mouse anti-p21 (A) and goat anti-p21 (C). Bars, 100 µm (B) and 20 µm (C).
(TIF)

Figure S4 ASF1a and HIRA are both required for SAHF formation. Triple staining for p21, DAPI and ASF1a or HIRA in C7 and C8 cells that were transfected with the indicated combinations of siRNAs (A, ASF1a; A2, ASF1a-2; H2, HIRA-2; H4, HIRA-4; C, control) 48 h prior to addition of 4OHT, which was then maintained for another 4 days. Both ASF1a and HIRA siRNAs significantly inhibited p53/p21-mediated SAHF formation in C7 and C8 cells. The arrows indicate cells with knockdown of ASF1a (top panel) or HIRA (bottom panel). The arrowheads indicate cells with SAHF. Antibodies used here included mouse anti-HIRA and goat anti-p21. Bars, 20 µm.
(TIF)

Figure S5 Verification of nucleolar ASF1a staining by ASF1a knockdown. IIICF/c cells were stained for ASF1a 3 days after transfection of ASF1a or control siRNA. Nucleolar ASF1a staining was substantially depleted in cells treated with ASF1a siRNA. Bar, 20 µm.
(TIF)

Figure S6 Nucleolar localization of ASF1b is less prominent than ASF1a. Proliferating C7 cells were double stained for B23 and ASF1a or ASF1b. Nucleolar ASF1b staining was less prominent than that of ASF1a. Antibodies used here included rabbit anti-ASF1b mAb. Bar, 20 µm.
(TIF)

Table S1 Proportion of SAHF-positive C7 and C8 cells after treatment with siRNAs and 4-hydroxytamoxifen (4OHT).
(DOC)
