# Supplementary figures and images for: HP1-Mediated Formation of Alternative Lengthening of Telomeres-Associated PML Bodies Requires HIRA but Not ASF1a
Source: PLoS One. 2011 Feb 15;6(2):e17036. doi: 10.1371/journal.pone.0017036 (PMC3039646; doi:10.1371/journal.pone.0017036)

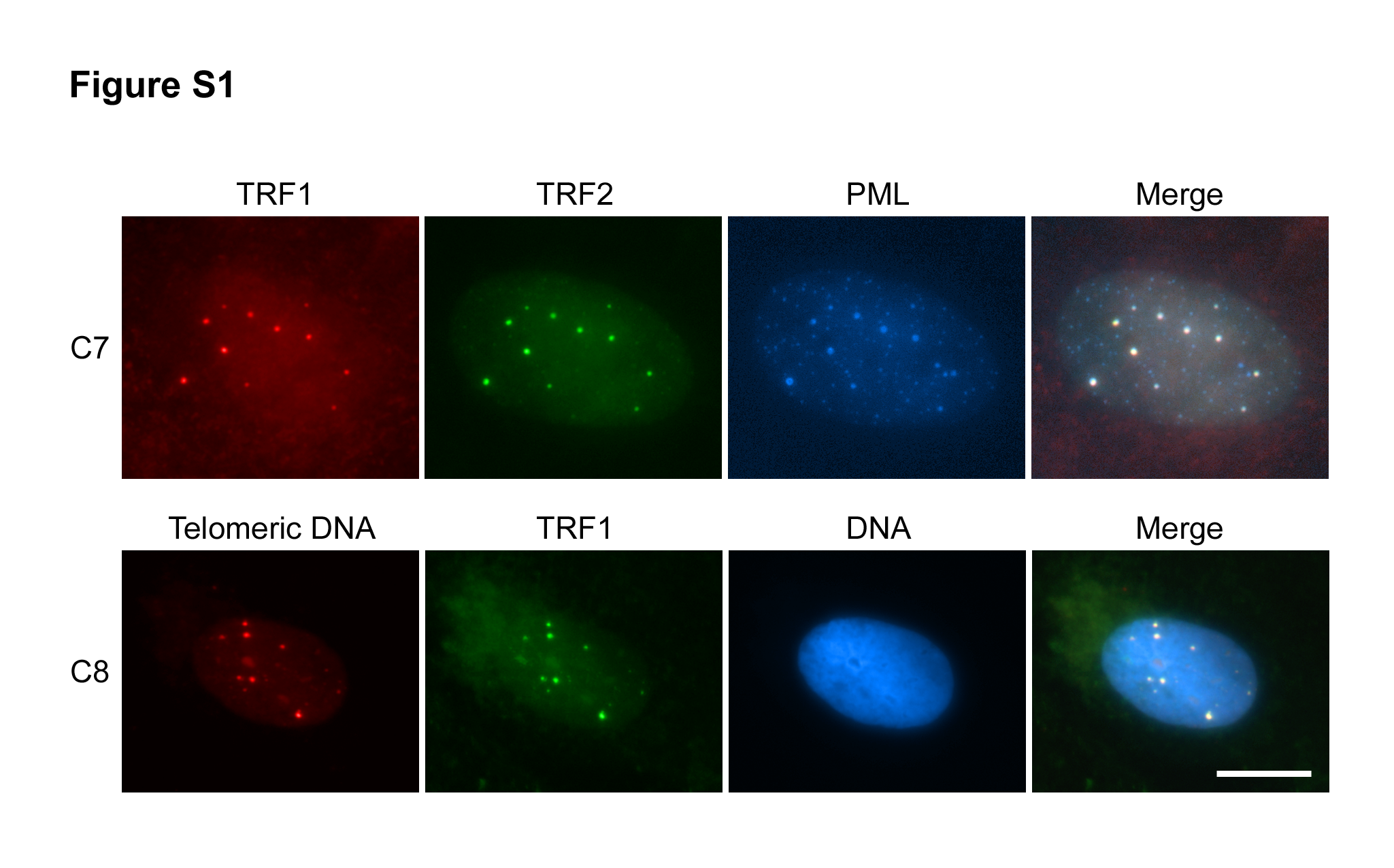

Supplement: Figure S1 — APBs can be detected interchangeably by either telomeric FISH or immunostaining of TRF1 or TRF2. After 4 days of 4OHT-treatment, APBs were induced in C7 and C8 cells, where colocalization was observed between prominent TRF1 or TRF2 foci and large PML bodies (top row), and between telomeric DNA and TRF1 foci (bottom row). The antibodies used for this supplementary figure and the subsequent figures are described in Materials and Methods and are only specified in the figure legends when Materials and Methods lists more than one antibody against that protein; here the goat anti-PML antibody was used. Bar, 20 µm. (TIF) [file pone.0017036.s001.tif]

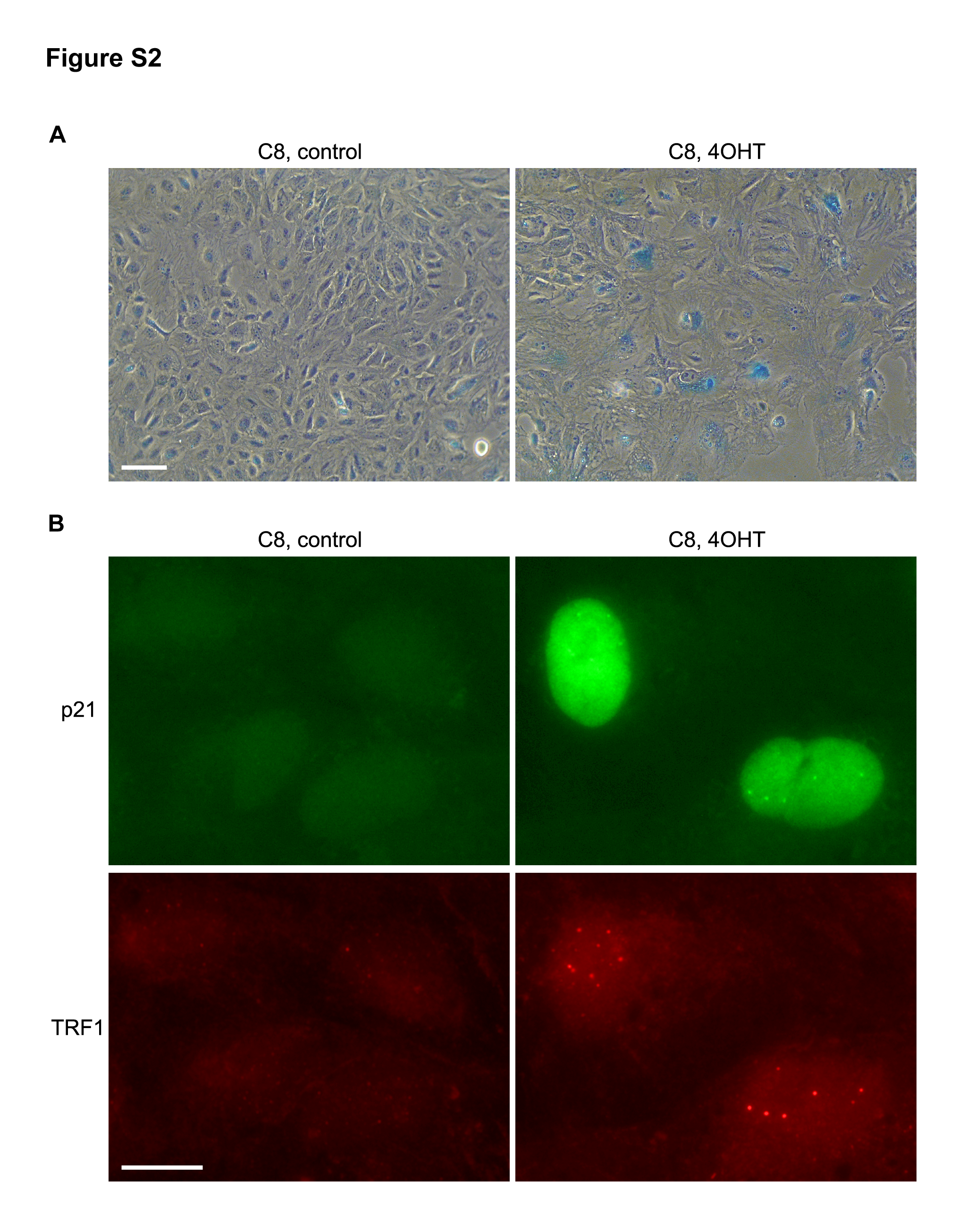

Supplement: Figure S2 — Induction of APBs is associated with p53/p21-mediated senescence. (A) SA-β-gal staining of C8 cells treated with 4OHT or ethanol vehicle for 4 days. SA-β-gal expression was found in 4OHT-treated C8 cells. (B) Double immunostaining of TRF1 and p21 in C8 cells treated with 4OHT or ethanol vehicle for 4 days. Large TRF1 foci were induced in cells positive for p21. Antibodies used here included mouse anti-p21 (B). Bars, 100 µm (A) and 20 µm (B). (TIF) [file pone.0017036.s002.tif]

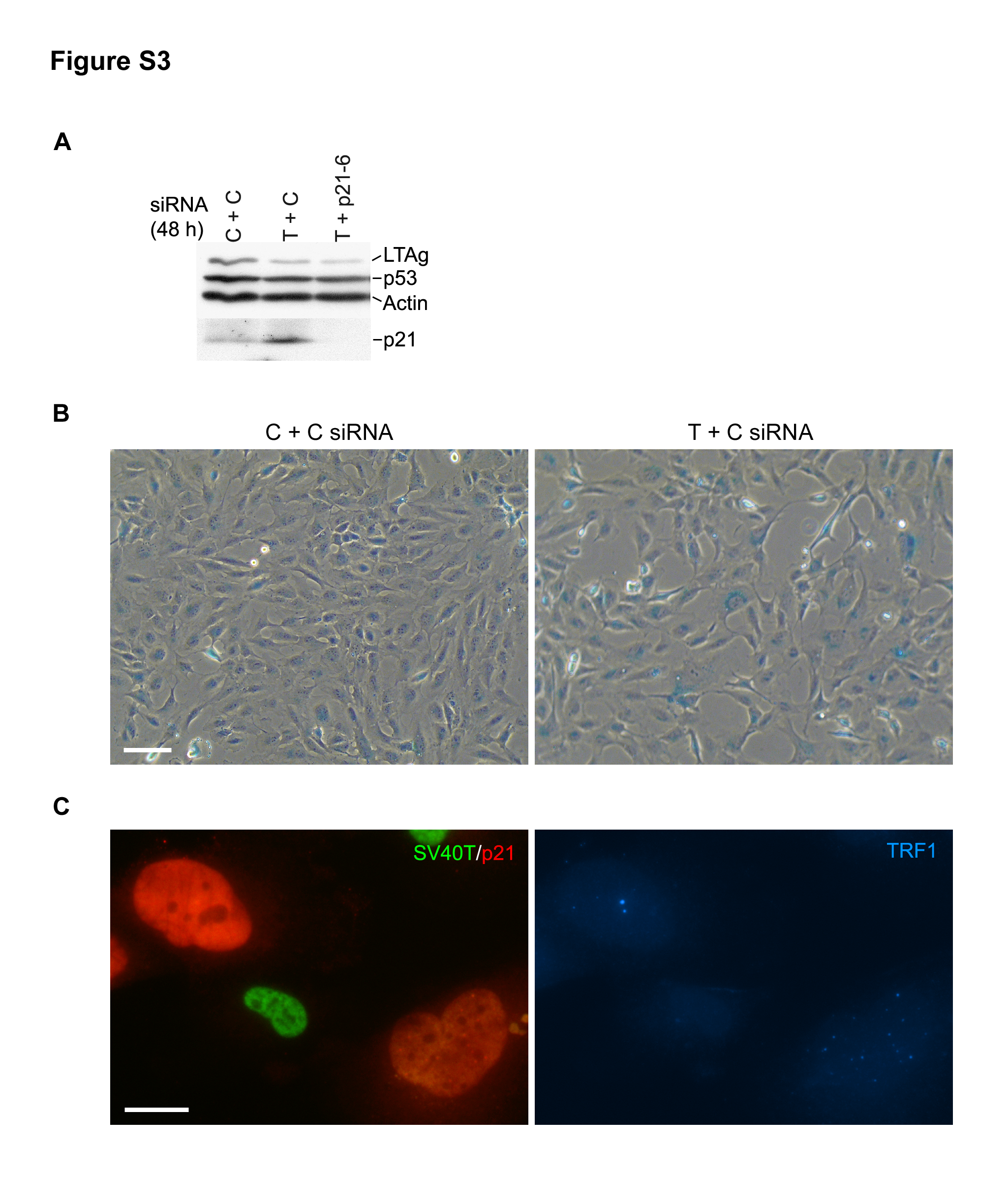

Supplement: Figure S3 — Induction of APBs in SV40-immortalized ALT cells by siRNA-mediated knockdown of LTAg. (A) Western blots showed induction of p21 in IIICF-T/B3 cells 2 days after transfection of SV40T and control (T+C) siRNAs. This induction of p21 was effectively abrogated by p21 siRNA (p21-6). The blots were probed with the indicated antibodies. (B) SA-β-gal staining of IIICF-T/B3 cells treated with the indicated combinations of siRNAs for 3 days. Strong SA-β-gal expression was found in cells treated with T+C siRNAs. (C) IIICF-T/B3 cells were triple stained for SV40T, p21 and TRF1 4 days after SV40T siRNA transfection. APBs (visualized here as large TRF1 foci) were observed in cells with high levels of p21. Antibodies used here included mouse anti-p21 (A) and goat anti-p21 (C). Bars, 100 µm (B) and 20 µm (C). (TIF) [file pone.0017036.s003.tif]

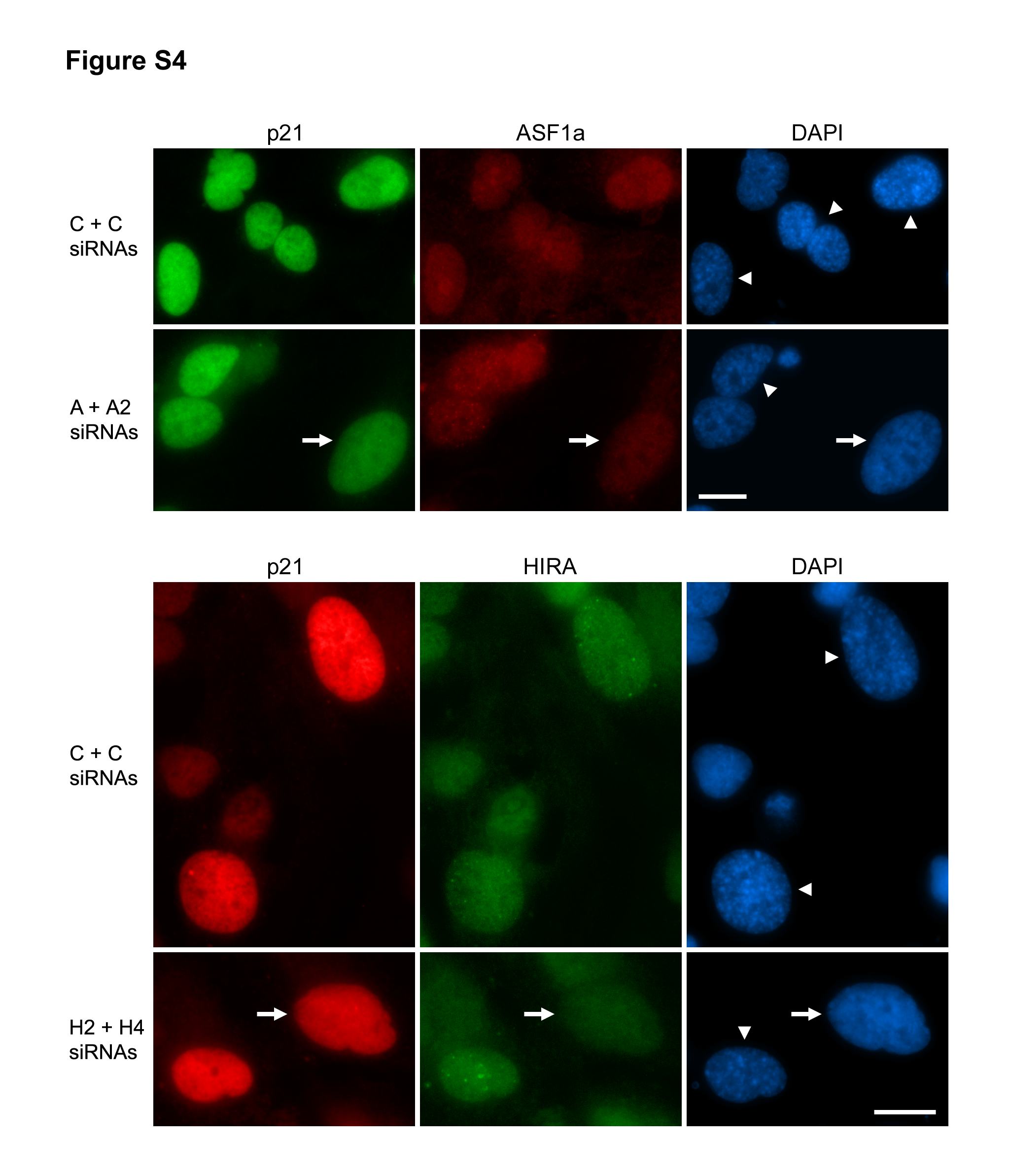

Supplement: Figure S4 — ASF1a and HIRA are both required for SAHF formation. Triple staining for p21, DAPI and ASF1a or HIRA in C7 and C8 cells that were transfected with the indicated combinations of siRNAs (A, ASF1a; A2, ASF1a-2; H2, HIRA-2; H4, HIRA-4; C, control) 48 h prior to addition of 4OHT, which was then maintained for another 4 days. Both ASF1a and HIRA siRNAs significantly inhibited p53/p21-mediated SAHF formation in C7 and C8 cells. The arrows indicate cells with knockdown of ASF1a (top panel) or HIRA (bottom panel). The arrowheads indicate cells with SAHF. Antibodies used here included mouse anti-HIRA and goat anti-p21. Bars, 20 µm. (TIF) [file pone.0017036.s004.tif]

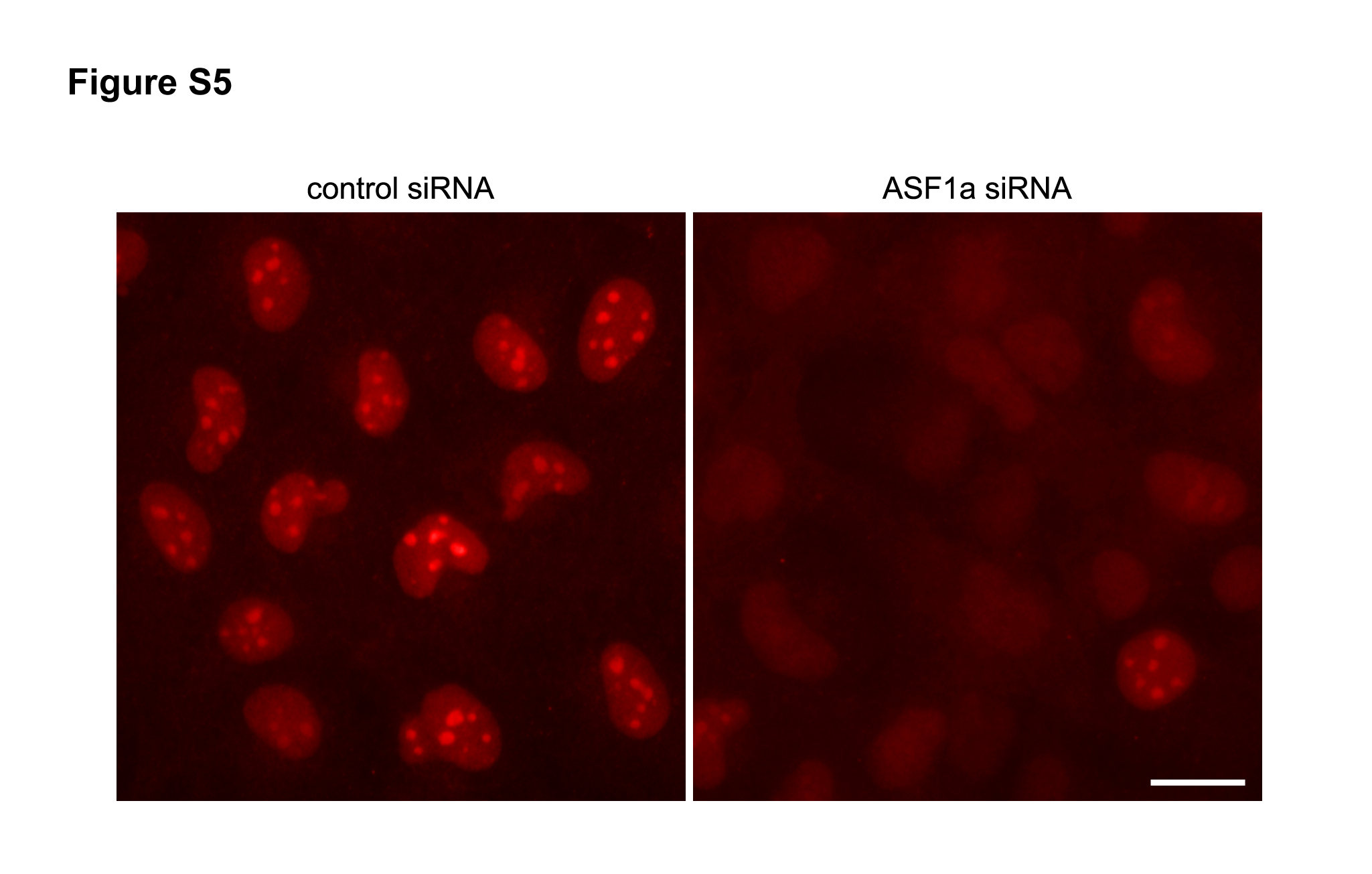

Supplement: Figure S5 — Verification of nucleolar ASF1a staining by ASF1a knockdown. IIICF/c cells were stained for ASF1a 3 days after transfection of ASF1a or control siRNA. Nucleolar ASF1a staining was substantially depleted in cells treated with ASF1a siRNA. Bar, 20 µm. (TIF) [file pone.0017036.s005.tif]

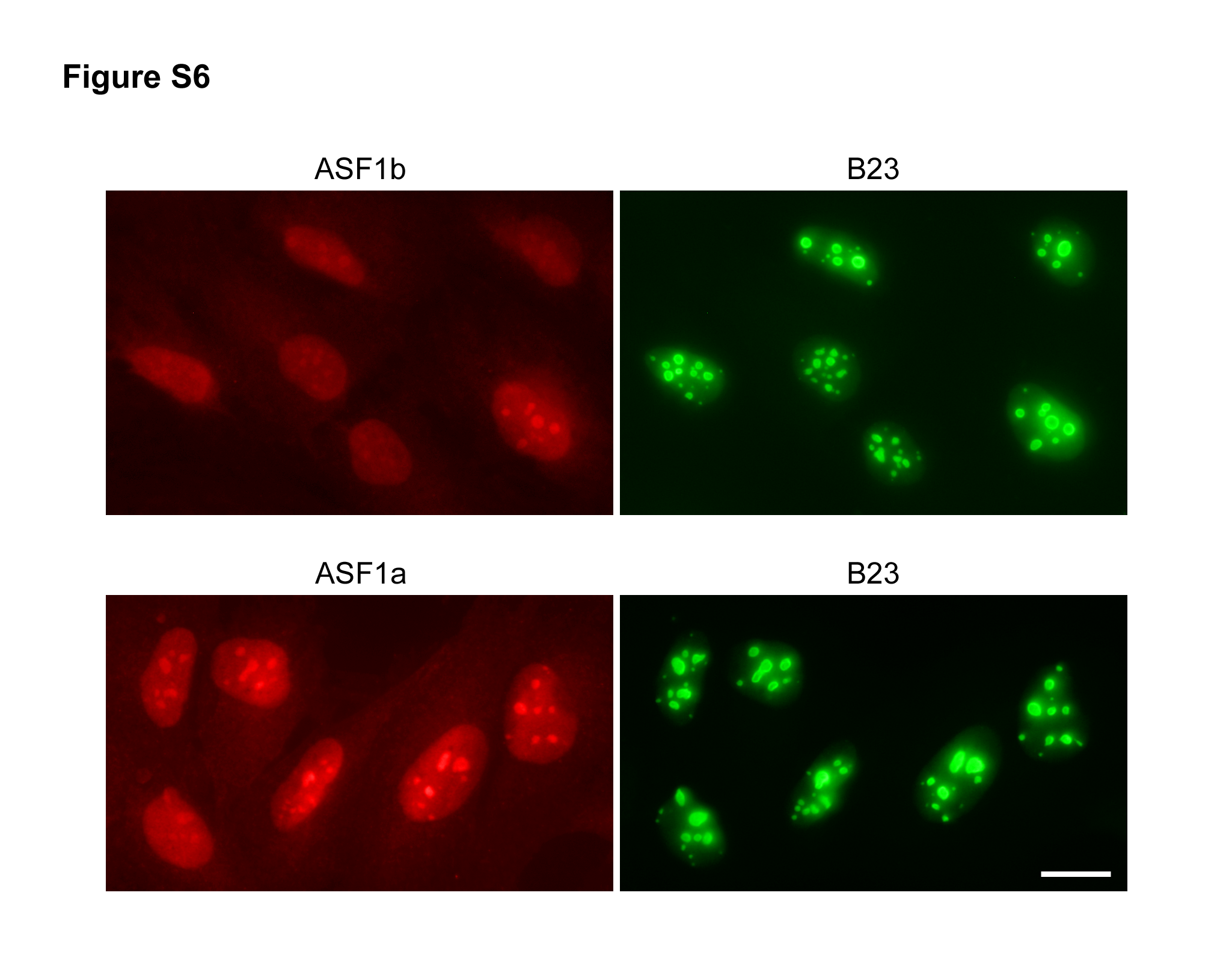

Supplement: Figure S6 — Nucleolar localization of ASF1b is less prominent than ASF1a. Proliferating C7 cells were double stained for B23 and ASF1a or ASF1b. Nucleolar ASF1b staining was less prominent than that of ASF1a. Antibodies used here included rabbit anti-ASF1b mAb. Bar, 20 µm. (TIF) [file pone.0017036.s006.tif]
