# Supplementary material for: HP1-Mediated Formation of Alternative Lengthening of Telomeres-Associated PML Bodies Requires HIRA but Not ASF1a
Source: PLoS One. 2011 Feb 15;6(2):e17036. doi: 10.1371/journal.pone.0017036 (PMC3039646; doi:10.1371/journal.pone.0017036)
Supplement: Table S1 — Proportion of SAHF-positive C7 and C8 cells after treatment with siRNAs and 4-hydroxytamoxifen (4OHT). (DOC) [file pone.0017036.s007.doc]

**Table S1** Proportion of SAHF-positive C7 and C8 cells after treatment with siRNAs and 4-hydroxytamoxifen (4OHT)

_________________________________________________________________

Cell lines siRNA-treatment^a^ Total (p21+)^b^ SAHF+ (%)

_________________________________________________________________

C7 C + C 280 59 (21.1)

C7 ASF1a + ASF1a-2 286 27 (9.4)

C8 C + C 269 42 (15.6)

C8 ASF1a + ASF1a-2 272 20 (7.4)

C7 C + C 263 50 (19.0)

C7 HIRA-2 + HIRA-4 266 21 (7.9)

C8 C + C 294 48 (16.3)

C8 HIRA-2 + HIRA-4 289 25 (8.7)

__________________________________________________________________

^a^Cells were transfected with 10 nM siRNA 48 h prior to the addition of 1 μM 4OHT, which was maintained for another 4 days. C, non-silencing control siRNA.

^b^Only cells that were positive by immunostaining for p21 were examined for SAHF.
